# PLCB4 copy gain and PLCß4 overexpression in primary gastrointestinal stromal tumors: Integrative characterization of a lipid-catabolizing enzyme associated with worse disease-free survival

**DOI:** 10.18632/oncotarget.15306

**Published:** 2017-02-13

**Authors:** Chien-Feng Li, Ting-Ting Liu, I-Chieh Chuang, Yen-Yang Chen, Fu-Min Fang, Ti-Chun Chan, Wan-Shan Li, Hsuan-Ying Huang

**Affiliations:** ^1^ Department of Pathology, Chi-Mei Medical Center, Tainan, Taiwan; ^2^ National Institute of Cancer Research, National Health Research Institutes, Tainan, Taiwan; ^3^ Department of Biotechnology, Southern Taiwan University of Science and Technology, Tainan, Taiwan; ^4^ Department of Pathology, Kaohsiung Chang Gung Memorial Hospital and Chang Gung University College of Medicine, Kaohsiung, Taiwan; ^5^ Division of Oncology, Department of Internal Medicine, Kaohsiung Chang Gung Memorial Hospital and Chang Gung University College of Medicine, Kaohsiung, Taiwan; ^6^ Department of Radiation Oncology, Kaohsiung Chang Gung Memorial Hospital and Chang Gung University College of Medicine, Kaohsiung, Taiwan; ^7^ Department of Pathology, Kaohsiung Medical University Hospital, Kaohsiung Medical University, Kaohsiung, Taiwan; ^8^ Bone and Soft Tissue Study Group, Taiwan Society of Pathology, Taiwan

**Keywords:** metabolism, lipid catabolism, PLCB4, transcriptome, GIST

## Abstract

To explore the implications of lipid catabolism-associated genes in gastrointestinal stromal tumors, we reappraised transcriptomic and genomic datasets and identified copy-gained and differentially upregulated *PLCB4* gene associated with tumor progression. On full sections, *PLCB4* mRNA abundance and PLCß4 immunoexpression were validated in 70 cases. On tissue microarrays, *PLCB4* gene copies and PLCß4 immunoexpression were both informative in 350 cases with *KIT/PDGFRA/BRAF* genotypes noted in 213. In GIST48 cell line, we stably silenced *PLCB4* and *YAP1* to characterize their functional effects and regulatory link. Compared with normal tissue, *PLCB4* mRNA abundance significantly increased across tumors of various risk levels (p<0.001), and was strongly correlated with immunoexpression level (p<0.001, r=0.468). Including polysomy (12.6%) and amplification (17.4%), *PLCB4* copy gain was detected in 105 (30%) cases and significantly more frequent (p<0.001) in cases exhibiting higher PLCß4 immunoexpression (82/175). Copy gain and protein overexpression were modestly associated with unfavorable genotypes (both p<0.05), strongly associated with increased size, mitosis, and risk levels defined by both the NIH and NCCN schemes (all p<0.001), and univariately predictive of shorter disease-free survival (both p<0.0001). In PLCß4-overexpressing cases, *PLCB4* copy gain still predicted worse prognosis (p<0.0001). In a multivariate comparison, both overexpression (p=0.007, hazard ratio: 2.454) and copy gain (p=0.031, hazard ratio: 1.892) exhibited independent impact. *In vitro*, YAP1 increased *PLCB4* mRNA and protein expression, and both molecules significantly promoted cell proliferation. Being driven by copy gain or YAP1, PLCß4 is a novel overexpressed enzyme regulating lipid catabolism that promotes cell proliferation and independently confers a worse prognosis.

## INTRODUCTION

The majority of gastrointestinal stromal tumors (GISTs) harbor mutually exclusive *KIT* or *PDGFRA* mutations, which drive tumor inception and dictate treatment response to imatinib [1]. Although the prognostic utility of NIH and NCCN risk schemes is effectual [2, 3], better characterization of molecular aberrations in tumor progression may refine prognostication of GISTs [4]. Clinical trials recently demonstrated the benefit of adjuvant imatinib therapy for moderate-risk and high-risk GISTs [5], which renders counseling regarding outcomes imperative, particularly in cases harboring imatinib-sensitive *KIT/PDGFRA* genotypes. In GISTs, secondary drug resistance to imatinib or other kinases-targeting agents inevitably emerges following initial responses [6], a challenge necessitating identification of novel non-kinase targets. It is desirable to characterize molecular aberrations of other signaling pathways, such as deregulated metabolism-associated enzymes that provide cellular energy and building blocks to sustain proliferation and metastasis of cancer cells [7] but remain largely unexplored in GISTs.

In this study, elucidation of lipid metabolism-associated enzymes was hypothesized to facilitate risk assessment. We began reappraising the transcriptomic datasets to search for metabolic driver genes involving the lipid catabolic process, which exhibited differential upregulation in high-risk and metastasizing GISTs at diagnosis with concordant copy number alterations (CNAs) in genomic profiling. This data-mining approach identified *PLCB4* as a top-ranking upregulated candidate with non-random copy gain in aggressive tumors. To the full extent of genetic, transcriptional, and translational characterization, we independently validated the clinical relevance of *PLCB4* copy gain and PLCß4 overexpression in GISTs. *PLCB4* encodes the ß4 isoform of phosphoinositide-specific phospholipase C (PLC) isoenzymes, a superfamily orchestrating the metabolism of inositol lipids [8]. The PLCß4 immunoexpression level had strong correlations with DNA copies and mRNA abundance. These genetic and expressive alterations exhibited robust associations with adverse clincopathological factors, unfavorable genotypes, and worse outcomes. Through polysomy or amplification, *PLCB4* copy gain represents one mechanism driving PLCß4 ovexpression and conferring biological aggressiveness, with strong negative prognostic impact in both the entire cohort and the PLCß4-overexpressing sub-cohort. Using RNA interference in a PLCß4-expressing GIST cell model, we functionally validated the pro-proliferative oncogenic attribute of *PLCB4* and its expression upregulated by increased YAP1, hence providing insights into alternative non-amplified mechanism(s) that may drive *PLCB4* expression.

## RESULTS

### Differential *PLCB4* mRNA upregulation and copy gain in aggressive GISTs

**Figure 1:**
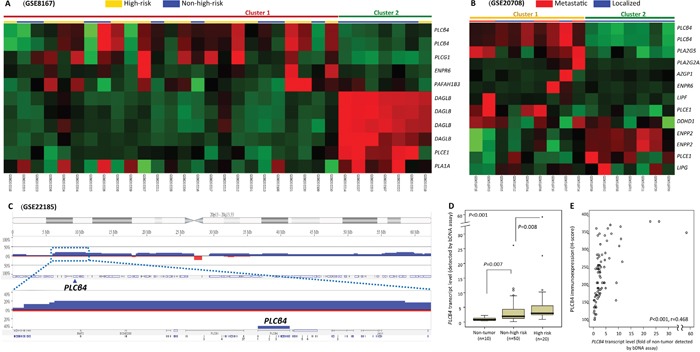
Transcriptomic and genomic reappraisals identify *PLCB4* as the top-ranking lipid catabolism-regulating gene associated with aggressive GISTs **A, B**. Heatmaps of unsupervised hierarchical clustering analysis for differentially expressed genes between non-high-risk and high-risk samples (A, GSE8167) and between localized and metastasizing GISTs (B, GSE20708) at diagnosis. In both comparisons, two each crudely segregated clusters were identified, which comprised more high-risk (yellow bars) and metastasizing (red bars) GITSs in the upper left quadrants, respectively. Listed on the right of individual heatmaps were differentially upregulated (*red*) and downregulated (*green*) genes involving the lipid catabolic process (GO: 0006629). **C**. Analysis of genomic profiling dataset in the public domain (GSE21185) reveals an amplicon at 20p12.2-12.3 harboring *PLCB4* gene, exhibiting copy gain in 20% (4 out of 20) GISTS. **D, E**. In the Quantigene assay (D), *PLCB4* mRNA was validated to be differentially upregulated across GISTs of various risk levels (informative n=70) as compared with the normal tissues. *PLCB4* mRNA also exhibits significant risk level-associated increment, implying its role in tumor progression. In the same set of 70 GISTs, the scattered plot (E) showed a strong correlation between the *PLCB4* mRNA level on the *X* axis and H-scores of PLCß4 immunoexpression on the *Y* axis (see representative images in Figure 2A).

Focusing on 142 probes covering 77 genes regulating lipid catabolic process, unsupervised hierarchic clustering was performed for two transcriptomic datasets of GISTs and crudely separated the samples into two clusters. These comprised 7 and 9 genes differentially expressed between high-risk and non-high-risk samples in GSE8167 (Figure 1A, Supplementary Table 1) and between localized and metastasizing samples in GSE20708 (Figure 1B, Supplementary Table 2), respectively. Notably, *PLCB4* represented the top-ranking upregulated candidate common to both datasets, exhibiting strong associations with high-risk category and metastasis (both p≤0.0001) and remarkable expression fold changes (log_2_ ratios: 1.1827–2.4364). In the genomic dataset (GSE21185), the *PLCB4* copy number was non-randomly gained in 20% of samples (Figure 1C). In this context, we selected *PLCB4* to validate its clinical relevance in two independent tumor cohorts.

### *PLCB4* mRNA abundance associated with risk categories and immunoexpression

To validate the findings of transcriptomic reappraisals by Quantigene *PLCB4* mRNA quantification, we preselected cases having concordant assignment of high-risk versus non-high-risk category using both NCCN and NIH criteria [2, 3]. The detection of *PLCB4* mRNA abundance was informative in 10 normal tissues and 70 primary GISTs. Another 16 tumors could not be analyzed because of degraded nucleic acids. These 70 informative GISTs, 49 gastric and 21 intestinal, were classified as 20 high-risk and 50 non-high-risk cases (Supplementary Table 3). Compared with normal tissues, *PLCB4* mRNA abundance was significantly higher in GISTs as a whole (p<0.001, Figure 1D) and also increased from normal tissue over the non-high-risk group (p=0.007) to high-risk GISTs (p=0.008), indicating its implication in tumor progression. Notably, mRNA and protein levels of PLCB4 were strongly and positively associated with each other (p<0.001, r=0.468, Figure 1E).

### *PLCB4* copy gain and PLCß4 overexpression associated with each other, adverse clinicopathological factors, and unfavorable RTK genotypes

To characterize the clinical relevance of *PLCB4*, a TMA validation set was employed to evaluate gene copies and immunoexpression, yielding 350 GISTs informative for both variables and follow-up data (Tables 1, 2). These comprised 88 none- or very low-risk, 100 low-risk, 65 moderate-risk, and 97 high-risk cases based on NCCN guidelines, corresponding to 127 very low/low-risk, 110 intermediate-risk, and 113 high-risk cases according to NIH risk scheme (Supplementary Table 4). Among 213 GISTs previously analyzed for *KIT* and *PDGFRA* mutations, 22 cases harboring normal *KIT/PDGFRA* genotypes were confirmed to retain expression of SDHA and SDHB (Supplementary Figure 1A) and wild-type *BRAF* gene (Supplementary Figure 1B). The median value of averaged PLCß4 immunohistochemical H-scores was 245 (range, 100–390) and used to dichotomize overexpressed and under-expressed groups (n=175 each) (Figure 2A). The overexpressed group strongly correlated with increased *PLCB4* copies (Figure 2B) detected in 105(30%) cases (p<0.001), including polysomy in 44(12.6%) and amplification in 61(17.4%). The vast majority (59/61) of amplified cases exhibited PLCß4 overexpression, which was observed only in a half (23/44) of polysomic cases. However, more than a half (53.1%, 93/175) of PLCß4-overexpressing tumors did not display *PLCB4* copy gain by FISH, suggesting that alternative mechanism(s) may drive PLCß4 overexpression in GISTs. Increased *PLCB4* copies and PLCß4 overexpression were modestly associated with unfavorable genotypes (both p<0.05) and strongly related to increased size, mitosis, and risk levels defined by both NIH and NCCN schemes (all p<0.001, Table 1). However, non-gastric locations and presence of epithelioid histology were only associated with overexpression (p=0.022) and copy gain (p<0.001), respectively.

**Table 1:** Associations of PLCB4 expression and gene dosage with various clinicopathological parameters in 350 GIST patients

	PLCB4 Expression		p-value	*PLCB4* Gene Dosage		p-value
	Low Exp.	High Exp.		No gain	Polysomy & Amp.	
**Sex**			0.748			0.363
Male	87	90		125	57	
Female	88	85		120	48	
**Age (years)**	58.9±13.70	60.9±11.70	0.137	59.9±13.14	59.7±11.90	0.900
**Location**			0.022*			0.082
Gastric	116	95		155	56	
Non-gastric	59	80		90	49	
**Histologic Type**			0.802			<0.001*
Spindle	134	132		200	66	
Epithelioid & Mixed	41	43		45	39	
**Tumour Size (cm)^&^**	5.4±3.85	7.4±4.35	<0.001*	5.4±3.47	8.6±4.93	<0.001*
**Mitotic Count (50HPFs)^&^**	6.5±18.94	12.0±26.95	0.001*	5.3±12.24	18.3±37.01	<0.001*
**NIH Risk**			<0.001*			<0.001*
Low/Very low	85	42		116	11	
Intermediate	54	56		81	29	
High	36	77		48	65	
**NCCN Guideline**			<0.001*			<0.001*
None/Very low	66	22		82	6	
Low	50	50		81	19	
Moderate	32	33		40	25	
High	27	70		42	55	
**Mutation Type**			0.033*			0.023*
Favorable Type	54	52		78	28	
Unfavorable Type	39	68		63	44	
**PLCB4 Expression**						<0.001*
Low Exp.				152	23 (21^#^, 2^$^)	
High Exp.				93	82 (23^#^, 59^$^)	

^*^: Statistically significant, ^&^: Wilcoxon rank-sum test, #, $: numbers of polysomic and amplified cases, respectively.

**Table 2:** Univariate and multivariate analyses for disease-free survival according to *PLCB4* gene status, PLCß4 expression status, NCCN guidelines, and other prognostic factors

Parameter	Univariate analysis			Multivariate analysis		
	No. Case	No. Event	p-value	HR	95% CI	p-value
**Sex**			0.4667			
Male	177	43				
Female	173	44				
**Age (years)**			0.0584			
<70	259	59				
>=70	91	28				
**Location**			0.0023*			0.385
Gastric	211	40		1	-	
Non-gastric	139	47		0.798	0.480-1.328	
**Histologic Type**			<0.0001*			0.001*
Spindle	266	51		1	-	
Mixed/Epithelioid	84	36		2.364	1.408-3.971	
**Tumour Size (cm)^#^**			<0.0001*			
=<5 cm	161	16				
>5; =<10 cm	131	38				
>10 cm	58	33				
**Mitotic Count (50HPFs)^#^**			<0.0001*			
0-5	249	33				
6-10	43	14				
>10	58	40				
**NCCN Guideline**			<0.0001*			<0.001*
None/Very low	88	3		1	-	
Low	100	10		2.461	0.519-11.675	
Moderate	65	15		2.566	0.541-12.173	
High	97	59		8.408	1.913-36.963	
**Mutation Type**			0.0005*			0.091
Favorable type	106	22		1	-	
Unfavorable type	107	45		1.584	0.929-2.701	
**PLCß4 expression**			<0.0001*			0.007*
Low Expressed	175	20		1	-	
High Expression	175	67		2.454	1.274-4.730	
***PLCB4* gene status**			<0.0001*			0.031*
No gain	245	31		1	-	
Polysomy & Amp.	105	56		1.892	1.062-3.370	

#, Tumour size and mitotic activity were not introduced in multivariate analysis, since these two parameters were component factors of NCCN risk scheme; *, Statistically significant. HR, hazard ratio.

**Figure 2:**
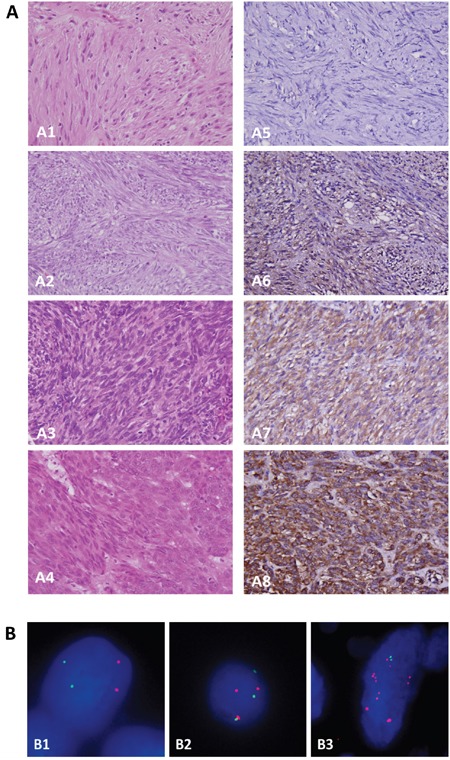
Independent validation for clinical implications of PLCß4 overexpression and *PLCB4* copy gain on tissue microarrays **A**. In one each representative GISTs classified as very low- (A_1_), low- (A_2_), intermediate- (A_3_), and high-risk (A_4_) levels, tumor cells exhibit no (A_5_), weak (A_6_), moderate (A_7_) to diffuse strong (A_8_) cytoplasmic reactivity to PLCß4, respectively. **B**. Using the probe directed against the centromeric sequence (*green*) as the reference, a locus-specific FISH probe targeting *PLCB4* gene (*red*) demonstrated normal status (B_1_), polysomy (B_2_), and amplification (B_3_) in one each representative GIST.

### Copy gain-driven PLCß4 overexpression was predictive of worst prognosis

**Figure 3:**
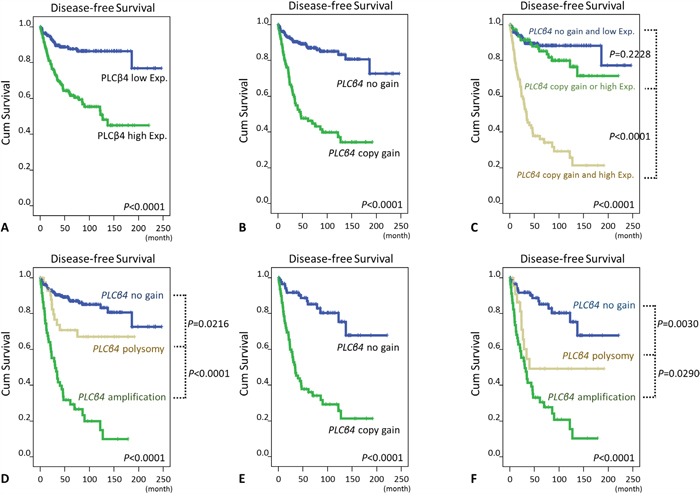
Kaplan-Meier analyses of univariate disease-free survival In all 350 informative cases, survival curves were shown according to the presence or absence of PLCß4 overexpression **A**. and *PLCB4* copy gain **B**., both aberrations combined versus neither or either of both **C**., and the *PLCB4* gene copy number status **D**. In 175 PLCß4-overexpressing cases, *PLCB4* copy gain **E**. and the *PLCB4* gene copy number status **F**. also significantly distinguished GISTs with different outcomes.

At the univariate level, *PLCB4* copy gain and PLCß4 overexpression were robustly predictive of shorter DFS (Figure 3A, 3B, Table 2, Supplementary Table 4, both p<0.0001) for the entire cohort. Moreover, GISTs exhibiting both copy gain and overexpression fared significantly worse than the counterparts featuring either one or none of both aberrations (Figure 3C, p<0.0001), while the latter two groups were not prognostically different. Notably, difference in *PLCB* gene status also exhibited strong prognostic impact (Figure 3D, p<0.0001) wherein the most unfavorable DFS predictor was amplification (p<0.0001, vs. polysomy), followed by polysomy (p=0.0216, vs. normal status). Even if only PLCß4-overexpressing GISTs were considered, DFS was still negatively related to *PLCB4* copy gain (Figure 3E, p<0.0001) and increased *PLCB* gene copy numbers (Figure 3F, p<0.0001), with significant differences in amplification vs. polysomy (p=0.0290) and in polysomy vs. normal status (p=0.0030). These clearly indicated the clinical relevance of determining the actual gene status in PLCß4-overexpressing cases.

Regarding the factor of risk levels, we separately evaluated the prognostic impact of either NCCN guidelines or NIH scheme in two multivariate regression models. When NCCN guidelines were incorporated (Table 2), both *PLCB4* copy gain (p=0.031, hazard ratio: 1.892) and PLCß4 overexpression (p=0.007, hazard ratio: 2.454) remained independently prognostic of worse outcomes, together with higher NCCN risk levels and presence of epithelioid histology (both p<0.001). However, unfavorable genotypes only exhibited a trend toward marginal significance. When NIH scheme, rather than NCCN criteria, was considered, only PLCß4 protein overexpression remained prognostically significant (p=0.001, Supplementary Table 4), together with higher NIH risk levels and presence of epithelioid histology (both p<0.001). However, *PLCB4* copy gain and unfavorable genotypes were not prognostically independent.

### Pro-proliferative function of PLCß4 linked to the upregulation by YAP1

**Figure 4:**
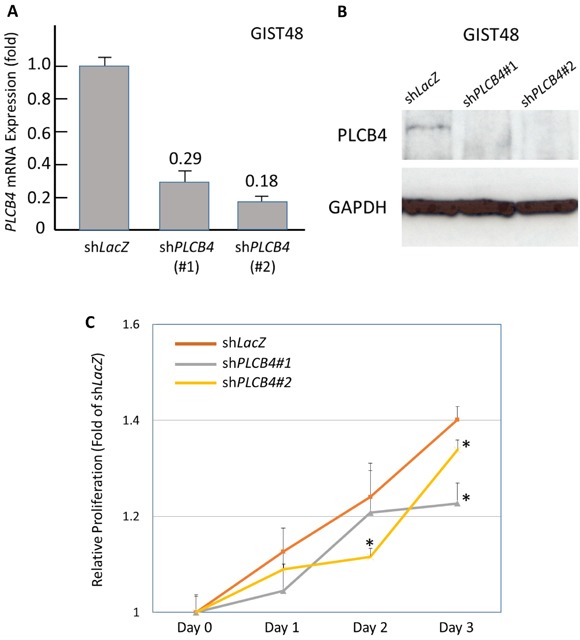
Stable RNA interference targeting *PLCB4* decreased cell proliferation Compared with *shLacZ*, both clones of short hairpin RNAs against *PLCB4*, s*hPCLB4#1 and shPLCB4#2*, remarkably decreased the mRNA and protein expression levels of PLCB4 in the PLCß4-expressing GIST48 cell line, as demonstrated by real-time RT-PCR **A**. and western blotting **B**. assays. When transduced with s*hPCLB4#1* and *shPLCB4#2*, the proliferation of GIST48 cells determined by the Brdu assay **C**. significantly declined from 72h or 48h onward, respectively.

**Figure 5:**
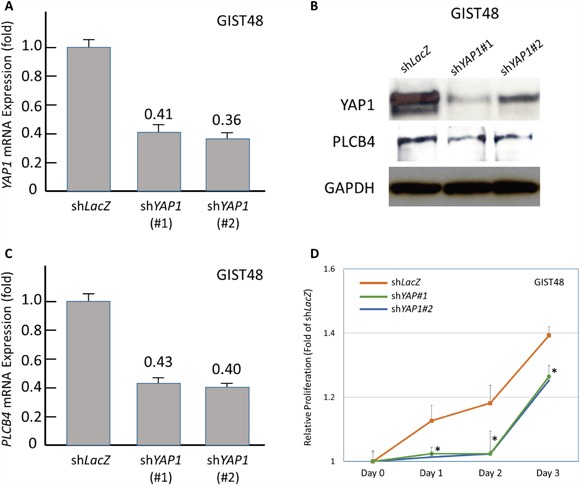
Stable RNA interference against YAP1 decreased PLCB4 expression and cell proliferation Compared with *shLacZ*, short hairpin RNAs against *YAP1*, s*hYAP1#1 and shYAP1#2*, effectively decreased the mRNA **A**. and protein **B**., (*upper row*) expression of YAP1 as determined by real-time RT-PCR and western blots, respectively. This genetic approach also caused significant decrease in the expression levels of PLCß4 protein (B, middle row) and *PLCB4* mRNA **C**. Moreover, the cell proliferation of GIST48 cells transduced with either clone of *shYAP1* **D**. exhibited remarkable decrease from 24h onward, compared with *shLacZ control*.

As stated above, *PCLB4* copy gain and PLCß4 overexpression both exhibited the strong prognostic negative impact on GISTs with discrepancy in their frequencies of aberrations. Accordingly, we used a PLCß4-expressing GIST48 cell line to explore the oncogenic function of PLCß4 in GISTs and its regulatory link to YAP1 as other possible non-amplified mechanism to increase expression in light of the recent discovery of involvement of PLCß4 in YAP1-active mesothelioma cell proliferation [9]. RNA interference was employed, and two *shPLCB4* clones achieved stable silencing as validated by both quantitative reverse-transcriptase polymerase chain reaction (RT-PCR) and western blotting assays (Figure 4A, 4B). Compared with *shLacZ*, both *shPLCB4* clones significantly decreased proliferative GIST48 cells from 48h or 72h onward (Figure 4C), hence confirming the proliferation-promoting attribute of PLCß4 in GISTs. Further, we stably knocked down YAP1 expression and observed remarkably decreased levels of mRNA and protein (Figure 5A, 5B), which significantly attenuated the expression of PLCß4 protein and *PCLB4* mRNA (Figure 5B, 5C), indicating a positive regulatory effect of YAP1 on *PCLB4* expression at the mRNA level. From Day 1 onward, stable YAP1 knockdown even more drastically decreased cell proliferation than *shPLCB4* (Figure 5D), implying that PLCß4 may represent only one of the downstream effectors of YAP1 in promoting cell proliferation.

## DISCUSSION

Among cancer-associated metabolic aberrations, accumulated CNAs attract increased attention for their dosage effects on the expression of lipid metabolism-regulating genes [10, 11]. Recently, an in silico study on glioblastomas indicated significant links between perturbed phosphoinositide metabolism and the CNAs of PLC isoenzymes-encoding genes, including amplified *PLCB4* among others [11]. To coordinate signals modulating cellular processes, various PLCs isoenzymes regulate phospholipid turnover at the cellular membrane though hydrolyzing phosphoinositides (e.g., phosphatidylinositol-4,5-diphospate) to generate diacylglycerol and inositol-1,4,5-trisphosphate as secondary messengers [12, 13]. Diacylglycerol mediates the activation of protein kinase C, while inositol-1,4,5-trisphosphate triggers calcium release from the intracellular reservoir [12, 13]. Notably, PLC025B and PLCγ may confer diverse cancer-promoting functions through deranging the balance among phosphoinositides-associated substrates, messengers, and signaling pathways [12, 13]. In mesenchymal tumors, R707Q mutation constitutively activates PLCγ1 and promotes evasion of apoptosis, migration, and invasiveness in angiosarcomas [14].

Of PLCß isoforms, overexpressed PLCß2 in breast carcinomas exhibits oncogenic functions and poor prognostic impact through promoting G2/M cell cycle progression and cell migration [15, 16]. Being abundant in the cerebellum and retina [17], PLCß4 contributes to the development of the first and second pharyngeal arches in the embryo, and germline *PLCB4* mutations occur in most patients with auriculocondylar syndrome, a rare craniofacial malformation manifesting mandibular deformity and characteristic “question-mark” ears [18]. However, the role of PLCß4 in cancer biology remains largely undefined, although a subset of uveal melanomas harbor a novel driver mutation at codon Asp630 [8]. In human cancers, *PLCB4* amplification has not been formally documented as a mechanism underlying overexpression, while it is provisionally catalogued in 20.6% of prostatic carcinomas exhibiting neuroendocrine phenotypes by the cBioPortal platform for cancer genomics [19].

As a proof of concept, we corroborated the clinical relevance of the increased dosage effect of *PLCB4* gene in human neoplasms for the first time, highlighting the presumable conversion of copy gain into upregulated mRNA and overexpressed protein in an aggressive subset of GISTs. This reasoning is based on the strong positive associations of PLCß4 immunoexpression levels with mRNA abundance and gene copy status in two independent cohorts. Compared with non-tumoral tissues, the *PLCB4* mRNA abundance was not only significantly higher in the whole group of tumors but also increased stepwise with risk increment. At the DNA and protein levels, PLCß4 overexpression and *PLCB4* copy gain both robustly characterized GISTs featuring increased tumor size, mitosis, and higher risk levels defined by both NIH and NCCN schemes. These strong links indicated that the oncogenic role of PLCß4 in GISTs is, at least in part, attributable to copy gain, displaying polysomy or amplification as a manifestation of genetic instability. Intriguingly, we found modest associations of unfavorable *KIT/PDGFRA/BRAF* genotypes with both *PLCB4* copy gain and PLCß4 overexpression, whereas it remains indefinite whether these findings indeed connote biological links.

Gene amplification is one mechanism underlying overexpression of various oncoproteins, while increased gene dosage secondary to polysomy is not clearly established for its clinical and biological implications in cancers [20]. Even if PLCß4 overexpression and *PLCB4* copy gain both predicted DFS, our results highlighted the clinical relevance of *PLCB4* copy gain, being potentially distinctive from protein overexpression. In both the entire cohort and PLCß4-overexpressing GISTs, there were significantly different DFS rates between copy-normal and polysomic cases and between polysomic and amplified cases. This finding resonated with the polysomy of chromosome 17 in breast carcinomas, which, to a lesser degree than *ERBB2* amplification, generally associates with clincopathological aggressiveness and worse prognosis [20]. Currently, there is lack of ample evidence to proclaim PLCß4 immunohistochemistry as a surrogate of *PLCB4* FISH assay. The latter is desirable to determine the gene status in PLCß4-overexpressing GISTs for prognostic reference and needful for the prospective follow-up in the *PLCB4*-polysomic cases. In the multivariate model incorporating NCCN risk levels, we substantiated that both PLCß4 overexpression and *PLCB4* copy gain were independent prognosticators, conceivably denoting that PLCß4 confers growth advantages and exasperates the progression of GISTs. However, *PLCB4* copy gain was detected only approximately a half (47%) of PLCß4-overexpressing tumors in our TMA cohort, indicating operation of alternative upregualtory mechanism(s). In this regard, we further demonstrated that PLCß4 contributed to cell proliferation in a PLCß4-expressing GIST cell line, and increased expression of YAP1, also exhibiting a pro-proliferative attribute, could upregulate *PLCB4* expression likely at the mRNA level.

Conclusively, we have corroborated PLCß4 as an oncogenic lipid-catabolizing enzyme in primary imatinib-naïve GISTs, which can be driven by amplification- or polysomy-derived copy gain or upregulated by increased YAP1. Given their strong associations with the risk increment, copy gain is presumably converted into upregulated mRNA and overexpressed protein, hence defining an aggressive subset of GISTs independently predicted by *PLCB4* copy gain and protein overexpression for shorter DFS. Copy gain and protein overexpression are not fully equivalent in prognostic relevance, for which performance of *PLCB4*-specfic FISH assay is warranted to complement PLCß4 immunostaining in GISTs. Further investigations on deregulated PLCß4 caused by gene copies versus YAP1-mediated upregulation may better delineate the clinical implications of perturbed phosphoinositides metabolism and open an alternative therapeutic avenue for imatinib-resistant, high-risk GISTs.

## MATERIALS AND METHODS

### Reappraisal of published genomic and transcriptomic datasets

Transcriptomic datasets in Gene expression Omnibus were reappraised for imatinib-naïve gastric and intestinal GISTs with varying clinical and histological aggressiveness. The raw CEL files were log_2_-transformed and integrated using Nexus Expression 3 software (BioDiscovery Hawthorne, CA) to analyze probe sets associated with the lipid catabolic process in Gene Ontology (GO: 0006629). Unsupervised comparative analysis was performed to identify genes differentially upregulated in high-risk (GSE8167) and metastasizing (GSE20708) GISTs profiled by Affymetrix Microarray (HG-U133_Plus_2), compared with their non-high-risk and localized counterparts at diagnosis, respectively. In the genomic dataset (GSE21185) profiled by Agilent (G4412A) CGH Microarray, the somatic CNAs were profiled to evaluate the frequencies of concordant copy gain or deletion on chromosomes harboring differentially expressed genes in transcriptomic reappraisal and to depict the zoom-in view of chromosome 20p where *PLCB4* is located. To delineate the breakpoints, gains and losses in significant CAN regions were defined as log_2_ ratios of ≧ +0.20 or ≦ -0.20, respectively. The ranking in the expression fold changes (requiring at least ≧ 0.1 fold in log_2_-transformed ratio), the power of statistical significance (p≤0.01 by Student-t test), and the concordance with DNA copy alterations in genomic profiling were considered for prioritizing genes for validation.

### Validation cohorts

The institutional review boards of Chang Gung Hospital approved this study (102-3911B) under local ethical regulations. To validate the transcriptomic analysis, Quantigene measurement for *PLCB4* mRNA abundance was performed on 86 formalin-fixed, primary GISTs, including 10 recent cases with adjacent non-tumoral tissue selected as the control. Of these, 70 informative cases were assessed for PLCß4 immunoexpression on full sections. Another independent cohort comprised 370 primary tumors resected before 2009, from which triplicate cores for each case had been previously assembled into tissue microarrays (TMA) [21]. TMA sections were recut for *PLCB4*-specfic fluorescent *in situ* hybridization (FISH) and PLCß4 immunohistochemistry, yielding 350 informative cases in both assays with 213 successfully genotypes for *KIT*/*PDGFRA* as previously reported [21]. In both cohorts, all cases were imatinib-naïve before disease relapse and tabulated for clinicopathological characteristics listed in Table 1 and Supplementary Table 1.

### Quantigene branched-chain DNA assay

This assay employs a sandwich nucleic acid hybridization to quantitate the mRNA abundance of housekeeping and target transcripts in tissue homogenates of formalin-fixed specimens [22]. Briefly, specific probes targeting *PLCB4* transcript were customized for detection by QuantiGene Multiplex 2.0 assay system (Affymetrix/Panomics). Oligonucleotides of the probe set were mixed with the lysed formalin-fixed tissues, and the mixture was added to a 96-well plate coated with capture probe oligonucleotides. Target RNA was captured and incubated overnight at 55°C with removal of unbound material using 300 μl wash buffer for 3 runs, followed by hybridization of DNA amplifier molecules and three additional washes after incubation every time. The dioxetane alkaline phosphatase substrate Lumiphos Plus was added to the reaction wells for detection by Luminex 100 microplate luminometer (Luminex). The detected readout of *PLCB4* mRNA abundance was further normalized by the expression level of reference *GAPDH* transcript.

### *PLCB4*-specific FISH

A bacterial artificial chromosome probe (RP11-252K6, Invitrogen), spanning *PLCB4* at 20p12.2, was labeled with SpectrumOrange. The Chromosome 20 Control probe targeting the centromeric region (#041015, Empire Genomics) was labeled with Green 5-Fluorescein dUTP essentially following the previously described method [23]. *PLCB4* copy number was assessed on 4-μm TMA sections by these locus-specific probes using a routine FISH protocol. The average numbers of red and green signals were determined by examining approximately 200 tumour cells in triplicate tissue cores for each specimen. Gene amplification was defined as a ratio of the gene probe signal to the control probe signal (i.e., red/green) exceeding 2.5. Polysomy was called when the average number of control green signals per nucleus was ≧ 3, with the red/green ratio being ≧ 1 and <2.5.

### Immunohistochemistry for PLCß4, SDHA, and SDHB

Whole block and TMA sections were microwave-heated to retrieve tissue antigen and incubated with the primary antibody against PLCß4 (1:50; Abcam), followed by detection with ChemMate EnVision kit (DAKO) as our previous works [24, 25, 26]. Blind to molecular and survival data, one pathologist (T.T.L) independently assessed PLCß4 cytoplasmic expression using the method of H-score [26, 27], as defined by the equation, Σ*P_i_* (*i* + 1) where *i* is the intensity of stained tumour cells (0-3+) and *Pi* is the percentage of stained tumour cells ranging from 0% to 100%. Regarding the TMA cohort, PLCß4 immunoexpression level was dichotomized into overexpressed and low-expressed groups, and the cutoff was defined as the median value of individual averaged triplicate H-scores of 350 informative cases. To screen GISTs with potential defects in the succinate dehydrogenase (SDH) complex, 22 GISTs harboring a normal *KIT/PDGFRA/BRAF* genotype were evaluated for immunoexpression of SDHA (1:750; Abcam) and SDHB (1:200; Abcam) on full sections (Supplementary Figure 1A).

### Mutation analysis of *KIT*, *PDGFRA*, and *BRAF* genes

We had previously genotyped 213 GISTs through DNA extraction, PCR amplification, direct sequencing of *KIT* exon 11, and denatured high performance liquid chromatography screening for exons 9, 13, and 17 of the *KIT* gene and exons 12 and 18 of the *PDGFRA* gene with confirmatory sequencing [21]. Following reported thermal conditions and primer pairs [28], *BRAF* gene was only sequenced for 22 GISTs retaining the wild-type *KIT* and *PDGFRA* genes, given that the concurrence of *BRAF* mutation with either *KIT* or *PDGFRA* mutation is extraordinary rare, if any, in GISTs [29, 30]. In fact, all these 22 cases revealed no mutation in the hotspot exon 15 that encompasses codon Val600 (Supplementary Figure 1A).

### Cell culture

GIST48 cell line, a kind gift from Professor Jonathan Fletcher, is known to harbor a primary homozygous V560D mutation in *KIT* exon11 and acquire a secondary heterozygous D820A mutation in *KIT* exon17 following imatinib therapy. As previously described [21], GIST48 cells were cultured in IMDM (Invitrogen) supplemented with 15% fetal bovine serum (FBS), 100 U/ml penicillin/streptomycin, and 4 mM L-glutamine (Invitrogen) at 37°C in 5% CO_2_.

### RNA interference

To establish stably silenced clones of GIST48 cell line with the short-hairpin RNAs against endogenously expressed *PLCB4* or *YAP1*, the lentiviral vectors were obtained from Taiwan National RNAi Core Facility, including pLKO.1-*shLacZ* (TRCN0000072223: 5′-TGT TCGCATTAT CCGAACCAT-3′), pLKO.1-*shPLCB4* (TRCN0000007012: 5′-CGCTGACATCAGATCACA AAT-3′ designated as *shPLCB4*#1; TRCN0000007013: 5′-CCTGAGATCAATCATACACAA-3′ as *shPLCB4*#2) and pLKO.1-*shYAP1* (TRCN0000107268: 5′-GACCAAT AGCTCAGATCCTTT-3′ as *shYAP1#1*; TRCN0000107 269: 5′-CGACCAATAGCTCAGATCCTT-3′ as *shYAP1#2*). Viruses were produced by transfecting HEK293 cells with the vectors above using Lipofectamine 2000. For viral infection, 3×10^6^ GIST48 cells were incubated with 8 ml lentivirus in the presence of polybrene, followed by puromycin selection for stable clones of lentivirus-transduced cells.

### Quantification of transcripts of *PLCB4* and *YAP1*

RNeasy Mini kit (Qiagen, Valencia, CA) was used to extract total RNAs from stable clones of GIST cell lines with lentiviral vectors bearing *shPLCB4, shYAP1*, or *shLacZ*. RNAs were further reverse-transcribed using SuperScript™ III First-Strand Synthesis System (Invitrogen, Carlsbad, CA) according to the manufacturers’ instructions. Real-time RT-PCR was performed using an ABI StepOnePlus™ System to quantify the expression levels of *PLCB4* and *YAP1* transcripts using pre-designed TaqMan assay reagents (Hs00168656_m1 for *PLCB4*, Hs00902712_g1 for *YAP1*, and *POLR2A* [a.k.a, RNA polymerase polypeptide A] Hs01108291_ml, Applied Biosystems, Foster City, CA). The obtained data were normalized by the expression of *POLR2A* housekeeping transcript. The relative expression fold of *PLCB4* or *YAP1* mRNA was then given by 2^− Δ ΔCp^, where Δ ΔC_T_ = ΔC_T_ (_sh*PLCB4* or sh *YAP1*_)- ΔC_T_ (_sh*LacZ*_), ΔC_T_ represented the C_T_ of *PLCB4* or *YAP1* subtracted from the C_T_ of *POLR2A*.

### Western blots

The western blotting assay was performed to evaluate the endogenous PLCß4 expression and the efficiency and consequent alteration of silencing PCLB4 and YAP1 in GIST48 cells. Cell lysates containing 25 μg protein were separated by 4-12% gradient NuPAGE gel (Invitrogen), transferred onto PVDF membranes (Amersham), and probed with antibodies against PLCß4 (1:1000, BD Biosciences), YAP1 (1:10000, abcam), or GADPH (1;3000, Chemicon). After incubation with the secondary antibody, proteins were visualized by the chemiluminescence system (Amersham).

### Bromodeoxyuridine (BrdU) assay to assess DNA synthesis

DNA synthesis was assessed using an enzyme-linked immunosorbent assay-based and colorimetric BrdU assay (Roche Diagnostics) as previously described [21]. GIST48 cells transduced with *shPLCB4, shYAP1*, or *shLacZ* control were plated into a 96-well plate at density of 3000 cells per well, and DNA synthesis was evaluated at 24, 48, and 72 h. The absorbance of the samples was measured using an ELISA reader (Promega) at 450 nm, with the absorbance at 690 nm as reference.

### Statistical analysis

For the full-sectioned samples, Mann-Whitney U test was performed to examine the difference in *PCLB4* mRNA abundance between adjacent normal tissue and GISTs and between high-risk and non-high-risk groups. Pearson correlation analysis was used to evaluate the association between mRNA abundance and protein immunoexpression. In the TMA validation set, we evaluated the associations of *PLCB4* copy number and PLCß4 immunoexpression with clinicopathological factors using the Chi-square and Wilcoxon rank-sum tests for categorical and continuous variables, respectively. Follow-up data were available for 350 cases as of April 2009 (median, 49.9 months; range, 1–247). The endpoint was disease-free survival (DFS), which would not be confounded by imatinib therapy for disseminated diseases. Based on previously prognostic correlations for DFS [21], genotypes of GISTs were dichotomized into the favorable and unfavorable groups. The former included (i) *PDGFRA* mutation involving exons 12 or 18, (ii) 3′ tandem insertion of *KIT* exon 11 with or without point mutation, and (iii) single point mutation of *KIT* exon 11. The unfavorable genotypes comprised (i) Ala502-Tyr503 insertion of *KIT* exon 9, (ii) wild type for *KIT, PDGFRA*, and *BRAF* genes without loss of SDHA and SDHB, and (iii) 5′ deletion of KIT exon 11 with or without point mutation. We used the log-rank test to compare univariate prognostic analyses. In the multivariate analyses, significant prognosticators with univariate p<0.05 were generally introduced, including either NIH scheme or NCCN guidelines, while size and mitosis, being component factors of risk stratification, were not incorporated in the multivariate comparisons.

## SUPPLEMENTARY MATERIALS FIGURES AND TABLES





## Abbreviations

GIST: gastrointestinal stromal tumor
NIH: National Institute of Health
NCCN: National Comprehensive Cancer Network
PLCB4: phospholipase C-ß4
