# Supplementary material for: PLCB4 copy gain and PLCß4 overexpression in primary gastrointestinal stromal tumors: Integrative characterization of a lipid-catabolizing enzyme associated with worse disease-free survival
Source: Oncotarget. 2017 Feb 13;8(12):19997–20010. doi: 10.18632/oncotarget.15306 (PMC5386739; doi:10.18632/oncotarget.15306)
Supplement: Supplementary file 1 [file oncotarget-08-19997-s001.pdf]

PLCB4 copy gain and PLCß4 overexpression in primary gastrointestinal stromal tumors: Integrative characterization of a lipid-catabolizing enzyme associated with worse disease-free survival

SUPPLEMENTARY FIGURE AND TABLES

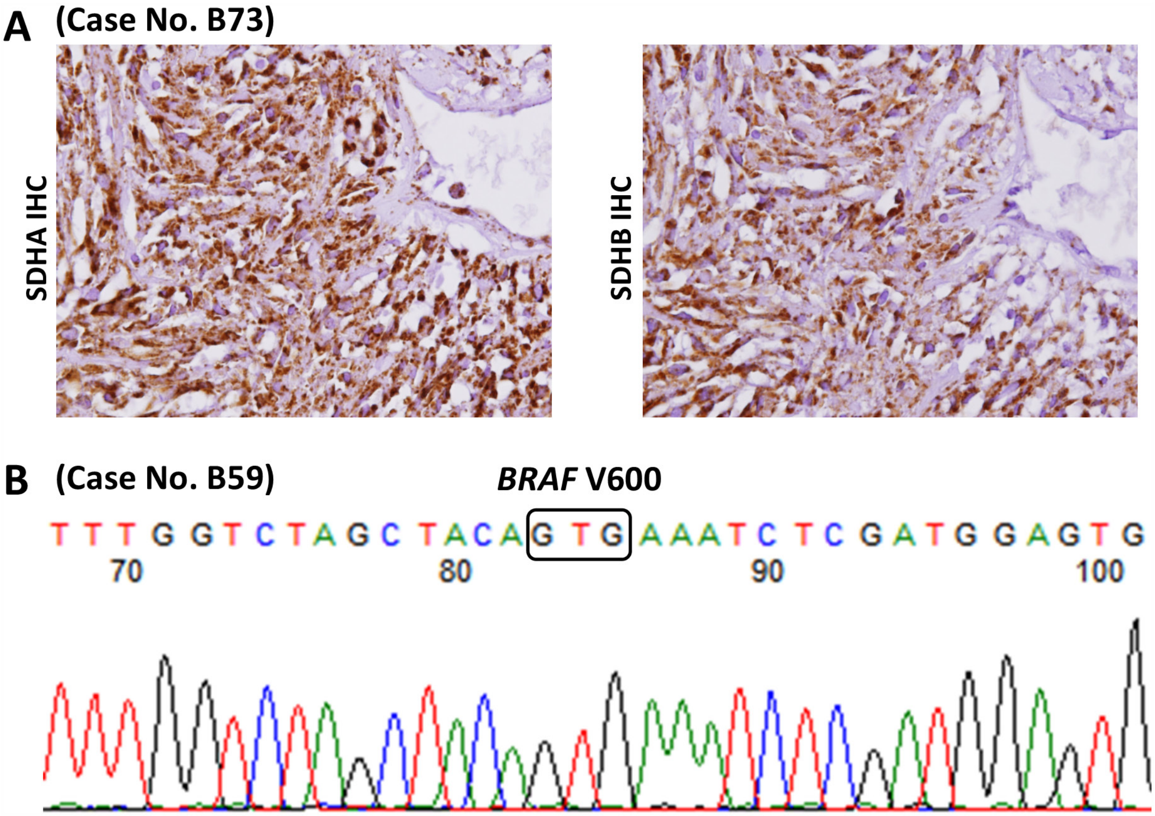

Supplementary Figure 1: Characterization of the expression status of mitochondrial SDH complex and *BRAF* mutation status in primary GISTs harboring wild-type *KIT* and *PDGFRA*. By using immunohistochemistry **A.**, one representative primary GIST (B73) exhibited preserved expression of SDHA (*left*) and SDHB (*right*). Sanger sequencing **B.** for another representative case (B59) demonstrated the wild-type nucleotide sequence of *BRAF* gene at exon 15.

Supplementary Table 1: Differentially expressed lipid catabolism-regulating genes (GO:0006629) associated with high risk level in the transcriptome of GISTs (GSE8167)

See Supplementary File 1

Supplementary Table 2: Differentially expressed lipid catabolism-regulating genes (GO:0006629) associated with metastasis in the transcriptome of GISTs (GSE20708)

See Supplementary File 1

Supplementary Table 3: Clinical and pathological variables of 70 GISTs informative in the Quantigene branched-chain DNA assay for quantitating *PLCB4* mRNA abundance

**Sex**	
Male	31
Female	39
**Age (years)**	61.0+/-10.95
**Location**	
Gastric	49
Non-gastric	21
**Histologic Type**	
Spindle	57
Epithelioid & Mixed	13
**Tumour Size (cm)**	5.4+/-3.33
**Mitotic Count (50 HPFs)**	6.3+/-11.31
**NIH Risk**	
Non-high-risk group	50 (Low/Very low risk, 21; Intermediate risk, 29)
High-risk group	20
**NCCN Guideline**	
Non-high-risk group	50 (None/Very low risk, 11; Low risk, 25; Moderate risk, 14)
High-risk group	20
**Mutation Type**	
Favorable Type	33
Unfavorable Type	37

HPFs, high power fields; NIH, National Institutes of Health; NCCN, National Comprehensive Cancer Network.

Supplementary Table 4: Univariate and multivariate analyses for disease-free survival according to *PLCB4* gene status, PLCß4 expression status, NIH criteria, and other prognostic factors

Parameter	Univariate analysis			Multivariate analysis		
	No. Case	No. Event	p-value	HR	95% CI	p-value
**Sex**			0.4667			
Male	177	43				
Female	173	44				
**Age (years)**			0.0584			
<70	259	59				
>=70	91	28				
**Location**			**0.0023***			0.769
Gastric	211	40		1	-	
Non-gastric	139	47		1.079	0.650-1.790	
**Histologic Type**			**<0.0001***			**0.001***
Spindle	266	51		1	-	
Mixed/Epithelioid	84	36		2.455	1.467-4.110	
**Tumour Size (cm)#**			**<0.0001***			
=<5 cm	161	16				
>5; =<10 cm	131	38				
>10 cm	58	33				
**Mitotic Count (50HPFs)#**			**<0.0001***			
0-5	249	33				
6-10	43	14				
>10	58	40				
**NIH Consensus**			**<0.0001***			**<0.001***
Very low/Low	127	6		1	-	
Intermediate	110	17		1.097	0.3794-3.173	
High	113	64		5.185	1.950-13.791	
**Mutation Type**			**0.0005***			0.088
Favorable type	106	22		1	-	
Unfavorable type	107	45		1.576	0.935-2.658	
**PLCB4 expression**			**<0.0001***			**0.001***
Low Exp.	175	22		1	-	
High Exp.	175	65		3.071	1.589-5.934	
***PLCB4* gene status**			**<0.0001***			0.108
No gain	245	31		1	-	
Polysomy & Amp.	105	56		1.602	0.901-2.850	

NCCN: National Comprehensive Cancer Network, HPF: high power filed, #, Tumour size and mitotic activity were not introduced in multivariate analysis, since these two parameters were component factors of NCCN guidelines; *, Statistically significant. HR, hazard ratio.
