# Supplementary material for: PLCB4 copy gain and PLCß4 overexpression in primary gastrointestinal stromal tumors: Integrative characterization of a lipid-catabolizing enzyme associated with worse disease-free survival
Source: Oncotarget. 2017 Feb 13;8(12):19997–20010. doi: 10.18632/oncotarget.15306 (PMC5386739; doi:10.18632/oncotarget.15306)
Supplement: Supplementary file 2 [file oncotarget-08-19997-s002.doc]

**Table-1. Differentially expressed lipid catabolism-regulating genes (GO:0006629) associated with high risk level in the transcriptome of GISTs (GSE8167)**

| **Probe** | **High vs. Non-high Risk** | | **Gene Symbol** | **Gene Name** | **Biological Process** | **Molecular Function** |
| --- | --- | --- | --- | --- | --- | --- |
|  | **Log ratio** | ***P*-value** |  |  |  |  |
| 203895_at | 1.2642 | <0.0001 | ***PLCB4*** | phospholipase C; beta 4 | intracellular signaling cascade, lipid catabolic process, lipid metabolic process, signal transduction | calcium ion binding, hydrolase activity, phosphoinositide phospholipase C activity, phospholipase C activity, protein binding, signal transducer activity |
| 203896_s_at | 1.1827 | 0.0001 | ***PLCB4*** | phospholipase C; beta 4 | intracellular signaling cascade, lipid catabolic process, lipid metabolic process, signal transduction | calcium ion binding, hydrolase activity, phosphoinositide phospholipase C activity, phospholipase C activity, protein binding, signal transducer activity |
| 202789_at | 0.7187 | 0.0008 | ***PLCG1*** | phospholipase C; gamma 1 | intracellular signaling cascade, lipid catabolic process, lipid metabolic process, signal transduction | calcium ion binding, hydrolase activity, phosphoinositide phospholipase C activity, phospholipase C activity, protein binding, receptor signaling protein activity, signal transducer activity |
| 203228_at | 0.5646 | 0.0016 | ***PAFAH1B3*** | platelet-activating factor acetylhydrolase; isoform Ib; gamma subunit 29kDa | cell proliferation, central nervous system development, lipid catabolic process, lipid metabolic process, nervous system development, regulation of progression through cell cycle, spermatogenesis | 1-alkyl-2-acetylglycerophosphocholine esterase activity, growth factor activity, hydrolase activity, hydrolase activity; acting on ester bonds, protein binding |
| 229916_at | 0.4605 | 0.0022 | ***ENPP6*** | ectonucleotide pyrophosphatase/phosphodiesterase 6 | choline metabolic process, lipid catabolic process, metabolic process, nucleotide metabolic process | glycerophosphodiester phosphodiesterase activity, hydrolase activity |
| 219584_at | -0.2733 | 0.0063 | ***PLA1A*** | phospholipase A1 member A | lipid catabolic process, lipid metabolic process | catalytic activity, hydrolase activity |
| 239904_at | -0.4484 | <0.0001 | ***PLCE1*** | Phospholipase C; epsilon 1 | G-protein signaling; coupled to IP3 second messenger (phospholipase C activating), Ras protein signal transduction, activation of MAPK activity, calcium-mediated signaling, cell proliferation, cytoskeleton organization and biogenesis, diacylglycerol biosynthetic process, elevation of cytosolic calcium ion concentration, epidermal growth factor receptor signaling pathway, heart development, inositol phosphate-mediated signaling, intracellular signaling cascade, lipid catabolic process, lipid metabolic process, phospholipid metabolic process, protein kinase C activation, regulation of G-protein coupled receptor protein signaling pathway, regulation of Ras protein signal transduction, regulation of cell growth, regulation of protein kinase activity, regulation of smooth muscle contraction, signal transduction, small GTPase mediated signal transduction | Ras GTPase binding, calcium ion binding, enzyme binding, guanyl-nucleotide exchange factor activity, hydrolase activity, phosphoinositide phospholipase C activity, phospholipase C activity, protein binding, receptor signaling protein activity, signal transducer activity |
| 225828_at | -0.8505 | <0.0001 | ***DAGLB*** | diacylglycerol lipase; beta | lipid catabolic process, lipid metabolic process | calcium ion binding, catalytic activity, hydrolase activity, metal ion binding, triacylglycerol lipase activity |
| 225832_s_at | -0.9725 | 0.0018 | ***DAGLB*** | diacylglycerol lipase; beta | lipid catabolic process, lipid metabolic process | calcium ion binding, catalytic activity, hydrolase activity, metal ion binding, triacylglycerol lipase activity |
| 226640_at | -1.0873 | 0.0002 | ***DAGLB*** | diacylglycerol lipase; beta | lipid catabolic process, lipid metabolic process | calcium ion binding, catalytic activity, hydrolase activity, metal ion binding, triacylglycerol lipase activity |
| 225833_at | -1.1187 | 0.0066 | ***DAGLB*** | diacylglycerol lipase; beta | lipid catabolic process, lipid metabolic process | calcium ion binding, catalytic activity, hydrolase activity, metal ion binding, triacylglycerol lipase activity |

**Table-2. Differentially expressed lipid catabolism-regulating genes (GO:0006629) associated with metastasis in the transcriptome of GISTs (GSE20708)**

| **Probe** | **Meta. vs. Non-meta.** | | **Gene Symbol** | **Gene Name** | **Biological Process** | **Molecular Function** |
| --- | --- | --- | --- | --- | --- | --- |
|  | **Log ratio** | ***P*-value** |  |  |  |  |
| 203895_at | 2.4364 | <0.0001 | ***PLCB4*** | phospholipase C; beta 4 | intracellular signaling cascade, lipid catabolic process, lipid metabolic process, signal transduction | calcium ion binding, hydrolase activity, phosphoinositide phospholipase C activity, phospholipase C activity, protein binding, signal transducer activity |
| 203896_s_at | 2.3538 | 0.0001 | ***PLCB4*** | phospholipase C; beta 4 | intracellular signaling cascade, lipid catabolic process, lipid metabolic process, signal transduction | calcium ion binding, hydrolase activity, phosphoinositide phospholipase C activity, phospholipase C activity, protein binding, signal transducer activity |
| 203649_s_at | 2.0372 | <0.0001 | ***PLA2G2A*** | phospholipase A2; group IIA (platelets; synovial fluid) | lipid catabolic process, phospholipid metabolic process | calcium ion binding, calcium-dependent phospholipase A2 activity, hydrolase activity, metal ion binding, phospholipase A2 activity, protein binding |
| 225970_at | 1.453 | 0.0015 | ***DDHD1*** | DDHD domain containing 1 | lipid catabolic process | hydrolase activity, metal ion binding |
| 205111_s_at | 1.3167 | 0.0014 | ***PLCE1*** | phospholipase C; epsilon 1 | G-protein signaling; coupled to IP3 second messenger (phospholipase C activating), Ras protein signal transduction, activation of MAPK activity, calcium-mediated signaling, cell proliferation, cytoskeleton organization and biogenesis, diacylglycerol biosynthetic process, elevation of cytosolic calcium ion concentration, epidermal growth factor receptor signaling pathway, heart development, inositol phosphate-mediated signaling, intracellular signaling cascade, lipid catabolic process, lipid metabolic process, phospholipid metabolic process, protein kinase C activation, regulation of G-protein coupled receptor protein signaling pathway, regulation of Ras protein signal transduction, regulation of cell growth, regulation of protein kinase activity, regulation of smooth muscle contraction, signal transduction, small GTPase mediated signal transduction | Ras GTPase binding, calcium ion binding, enzyme binding, guanyl-nucleotide exchange factor activity, hydrolase activity, phosphoinositide phospholipase C activity, phospholipase C activity, protein binding, receptor signaling protein activity, signal transducer activity |
| 205112_at | 1.2468 | 0.008 | ***PLCE1*** | phospholipase C; epsilon 1 | G-protein signaling; coupled to IP3 second messenger (phospholipase C activating), Ras protein signal transduction, activation of MAPK activity, calcium-mediated signaling, cell proliferation, cytoskeleton organization and biogenesis, diacylglycerol biosynthetic process, elevation of cytosolic calcium ion concentration, epidermal growth factor receptor signaling pathway, heart development, inositol phosphate-mediated signaling, intracellular signaling cascade, lipid catabolic process, lipid metabolic process, phospholipid metabolic process, protein kinase C activation, regulation of G-protein coupled receptor protein signaling pathway, regulation of Ras protein signal transduction, regulation of cell growth, regulation of protein kinase activity, regulation of smooth muscle contraction, signal transduction, small GTPase mediated signal transduction | Ras GTPase binding, calcium ion binding, enzyme binding, guanyl-nucleotide exchange factor activity, hydrolase activity, phosphoinositide phospholipase C activity, phospholipase C activity, protein binding, receptor signaling protein activity, signal transducer activity |
| 229916_at | 1.0712 | <0.0001 | ***ENPP6*** | ectonucleotide pyrophosphatase/phosphodiesterase 6 | choline metabolic process, lipid catabolic process, metabolic process, nucleotide metabolic process | glycerophosphodiester phosphodiesterase activity, hydrolase activity |
| 214159_at | 0.7227 | <0.0001 | ***PLCE1*** | Phospholipase C; epsilon 1 | G-protein signaling; coupled to IP3 second messenger (phospholipase C activating), Ras protein signal transduction, activation of MAPK activity, calcium-mediated signaling, cell proliferation, cytoskeleton organization and biogenesis, diacylglycerol biosynthetic process, elevation of cytosolic calcium ion concentration, epidermal growth factor receptor signaling pathway, heart development, inositol phosphate-mediated signaling, intracellular signaling cascade, lipid catabolic process, lipid metabolic process, phospholipid metabolic process, protein kinase C activation, regulation of G-protein coupled receptor protein signaling pathway, regulation of Ras protein signal transduction, regulation of cell growth, regulation of protein kinase activity, regulation of smooth muscle contraction, signal transduction, small GTPase mediated signal transduction | Ras GTPase binding, calcium ion binding, enzyme binding, guanyl-nucleotide exchange factor activity, hydrolase activity, phosphoinositide phospholipase C activity, phospholipase C activity, protein binding, receptor signaling protein activity, signal transducer activity |
| 215870_s_at | 0.5523 | <0.0001 | ***PLA2G5*** | phospholipase A2; group V | lipid catabolic process, phospholipid metabolic process | calcium ion binding, calcium-dependent phospholipase A2 activity, hydrolase activity, metal ion binding, phospholipase A2 activity |
| 219181_at | -0.2797 | 0.0050 | ***LIPG*** | lipase; endothelial | lipid catabolic process, lipid metabolic process | catalytic activity, heparin binding, hydrolase activity, lipoprotein lipase activity, phospholipase A1 activity, phospholipase activity, protein binding, triacylglycerol lipase activity |
| 239904_at | -0.4031 | 0.0001 | ***PLCE1*** | Phospholipase C; epsilon 1 | G-protein signaling; coupled to IP3 second messenger (phospholipase C activating), Ras protein signal transduction, activation of MAPK activity, calcium-mediated signaling, cell proliferation, cytoskeleton organization and biogenesis, diacylglycerol biosynthetic process, elevation of cytosolic calcium ion concentration, epidermal growth factor receptor signaling pathway, heart development, inositol phosphate-mediated signaling, intracellular signaling cascade, lipid catabolic process, lipid metabolic process, phospholipid metabolic process, protein kinase C activation, regulation of G-protein coupled receptor protein signaling pathway, regulation of Ras protein signal transduction, regulation of cell growth, regulation of protein kinase activity, regulation of smooth muscle contraction, signal transduction, small GTPase mediated signal transduction | Ras GTPase binding, calcium ion binding, enzyme binding, guanyl-nucleotide exchange factor activity, hydrolase activity, phosphoinositide phospholipase C activity, phospholipase C activity, protein binding, receptor signaling protein activity, signal transducer activity |
| 213222_at | -1.2155 | 0.0046 | ***PLCB1*** | phospholipase C; beta 1 (phosphoinositide-specific) | intracellular signaling cascade, lipid catabolic process, lipid metabolic process, regulation of progression through cell cycle, signal transduction | calcium ion binding, hydrolase activity, phosphoinositide phospholipase C activity, phospholipase C activity, signal transducer activity |
| 209392_at | -1.4405 | <0.0001 | ***ENPP2*** | ectonucleotide pyrophosphatase/phosphodiesterase 2 (autotaxin) | G-protein coupled receptor protein signaling pathway, cell motility, chemotaxis, lipid catabolic process, metabolic process, nucleotide metabolic process, phosphate metabolic process | alkylglycerophosphoethanolamine phosphodiesterase activity, endonuclease activity, hydrolase activity, metal ion binding, nucleic acid binding, nucleotide diphosphatase activity, phosphodiesterase I activity, transcription factor binding |
| 210839_s_at | -1.8995 | <0.0001 | ***ENPP2*** | ectonucleotide pyrophosphatase/phosphodiesterase 2 (autotaxin) | G-protein coupled receptor protein signaling pathway, cell motility, chemotaxis, lipid catabolic process, metabolic process, nucleotide metabolic process, phosphate metabolic process | alkylglycerophosphoethanolamine phosphodiesterase activity, endonuclease activity, hydrolase activity, metal ion binding, nucleic acid binding, nucleotide diphosphatase activity, phosphodiesterase I activity, transcription factor binding |
